# Chitosan Feasibility to Retain Retinal Stem Cell Phenotype and Slow Proliferation for Retinal Transplantation

**DOI:** 10.1155/2014/287896

**Published:** 2014-02-02

**Authors:** Girish K. Srivastava, David Rodriguez-Crespo, Amar K. Singh, Clara Casado-Coterillo, Ivan Fernandez-Bueno, Maria T. Garcia-Gutierrez, Joaquin Coronas, J. Carlos Pastor

**Affiliations:** ^1^Instituto Universitario de Oftalmobiología Aplicada (IOBA), Universidad de Valladolid, Campus Miguel Delibes Paseo de Belen 17, 47011 Valladolid, Spain; ^2^Centro en Red de Medicina Regenerativa y Terapia Celular de Castilla y León, 47011 Valladolid, Spain; ^3^Department of Chemical and Biomolecular Engineering, University of Cantabria, 39005 Santander, Spain; ^4^Instituto de Nanociencia de Aragon (INA), Universidad de Zaragoza, 50018 Zaragoza, Spain

## Abstract

Retinal stem cells (RSCs) are promising in cell replacement strategies for retinal diseases. RSCs can migrate, differentiate, and integrate into retina. However, RSCs transplantation needs an adequate support; chitosan membrane (ChM) could be one, which can carry RSCs with high feasibility to support their integration into retina. RSCs were isolated, evaluated for phenotype, and subsequently grown on sterilized ChM and polystyrene surface for 8 hours, 1, 4, and 11 days for analysing cell adhesion, proliferation, viability, and phenotype. Isolated RSCs expressed GFAP, PKC, isolectin, recoverin, RPE65, PAX-6, cytokeratin 8/18, and nestin proteins. They adhered (28 ± 16%, 8 hours) and proliferated (40 ± 20 cells/field, day 1 and 244 ± 100 cells/field, day 4) significantly low (*P* < 0.05) on ChM. However, they maintained similar viability (>95%) and phenotype (cytokeratin 8/18, PAX6, and nestin proteins expression, day 11) on both surfaces (ChM and polystyrene). RSCs did not express alpha-SMA protein on both surfaces. RSCs express proteins belonging to epithelial, glial, and neural cells, confirming that they need further stimulus to reach a final destination of differentiation that could be provided in *in vivo* condition. ChM does not alternate RSCs behaviour and therefore can be used as a cell carrier so that slow proliferating RSCs can migrate and integrate into retina.

## 1. Introduction

Retina is exposed over life to degenerative conditions. This leads into retinal dystrophies, followed by retinal diseases, and ultimately produces visual impairment [1]. Despite growing advances in retinal disease treatments, retinal diseases such as dry AMD, retinitis pigmentosa, and many others are still noncurable or need further improvements in treatment strategies. One of the main events of these diseases is loss of the retinal cells layers (RPE, photoreceptors, etc.) and their proper functions. These layers are crucial for maintaining retina anatomy and its functions in eye [2, 3].

From past few years, identification and characterization of stem cells of different origin have opened new avenues in cell replacement therapy [4, 5]. Retinal stem cells (RSCs) are present during embryonic development; they persist in quiescent forms in the adult mammalian eye in ciliary marginal zone [6–8]. Numerous reports showed that RSCs are promising for developing cell based treatments for retinal diseases [9, 10]. They have ability to differentiate into different retinal cell types such as RPE photoreceptors in appropriate differentiation conditions [6, 9]. Thus, RSCs could serve for replacing the damaged retinal layers in patients.

Cell transplantation, cell integration in tissue, and its proper function are still open issues of research. Different types of stem cells such as RSCs, neural stem cells (NSCs), bone marrow derived stem cells (BMSCs), and embryonic stem cells (ESCs) have achieved partial success in retinal transplantation studies [3, 11–13]. The reasons behind this partial success are various including poor viability and cell growth and loss of cell characteristics and functions. Cells integrate in the host's retina but they achieve partial success in establishing synaptic connections *in vivo*. These all studies demonstrated that an appropriate cell delivery system is crucial for retinal cell replacement therapy [14, 15].

Chitosan, poly[*β*(1→4)-2-amino-2-deoxy-D-glucopyranose], is a linear polysaccharide obtained by deacetylation of chitin, poly[*β*(1→4)-2-acetamido-2-deoxy-D-glucopyranose]. Chitin is an abundant natural polymer, comprised of repeating D-glucosamine units and it is produced from renewable sources, that is, the shell of crustaceans. Chitosan is very cheap, shows antibacterial and wound healing activities [16–19], and is approved by the Food and Drug Administration (FDA) for clinical application. It is used frequently in developing treatments based on nanomedicine and tissue engineering. In most of the tissue engineering studies, electrospined fibrous chitosan blended with other substances such as poly(*ε*-caprolactone) (PCL), polylactic (PLA), and polyglycolic (PGA) has been used to test the behaviors of different cell types such as mouse RSCs, mesenchymal cells [20, 21], and fibroblasts. However, these substances can induce inflammation due to elevated acidity during polymer hydrolysis. There may be local tissue degeneration. Processing difficulties may lead to inconsistent biodegradation rates and tissue response profiles or degradation profiles may not match the rate of tissue regeneration [22, 23]. This naturally occurring polymer, chitosan, as a cell delivery system offers options to overcome these problems because of their biocompatible and biodegradable nature, producing low toxic by-products on degradation. It has the possibilities to mold them in different formats such as porous scaffolds and can be incorporated into the different growth factors as TGF, BMP4, and so forth.

RSCs differentiation potential and chitosan characteristics encouraged us to investigate the feasibility of chitosan membrane (ChM) application in delivering retinal stem cells into retina.

## 2. Materials and Methods

### 2.1. Cell Culture

RSCs were isolated from porcine eyes following already published papers [9]. RSCs and proliferating clumps of RSCs (RSC spheres) were cultured in 25 cm^2^ flasks under standard culture conditions of 5% CO_2_ in the humidified air at 37°C. Medium was renewed at every 2-3-day interval. Standard culture medium for floating RSC spheres was DMEM/F12 supplemented with antibiotics penicillin (100 U/mL)-streptomycin (0.1 mg/mL), 1 mM sodium pyruvate, 2 mM L-glutamine and growth factors FGFb (20 ng/mL), EGF (20 ng/mL), heparin (2 *μ*g/mL), and B-27 (2% v/v) (Gibco, Invitrogen, Paisley, UK). Floating RSC spheres were dissociated after 24 days using 0.05% trypsin-tetrasodium ethylenediaminetetraacetate (Trypsin-EDTA, Gibco, Invitrogen, Paisley, UK), washed with phosphate-buffered saline (PBS; Gibco, Invitrogen, Paisley, UK), seeded and cultured in standard complete RSCs culture medium. This medium was prepared by supplementing 10% foetal bovine serum (FBS) in standard culture medium. Confluent RSCs layer was trypsinized, washed, and resuspended in PBS. Cell numbers and viability for cell seeding for each experiment were determined by standard trypan blue exclusion assay. ARPE19 cell line was purchased from the American Type Culture Collection (Manassas, VA, USA) and cultured in accordance with our published article [24] and used as a positive control.

### 2.2. Chitosan Membrane Preparation

ChMs were prepared and provided by INA-University of Zaragoza, Spain, for evaluating RSCs growth, viability, and characteristics. In brief, chitosan (Aldrich, high molecular weight) was dissolved in a 2 wt% acetic acid (Alfa Aesar, glacial) aqueous solution by stirring for 24 hours at 80°C. After filtration, 2.3 mL of chitosan 1 wt% solution was cast on PS Petri dishes and was evaporated at room temperature (RT) for 2 days. The 10 *μ*m-thick transparent membranes were treated in vacuum oven at 120°C for 24 hours to remove residual solvent and acid from the matrix prior to seeding RSCs.

### 2.3. RSCs Seeding on Chitosan Membrane

Each experiment was performed in 8-well chamber slides (8 mm × 8 mm), of which four were covered with ChM pieces of size 6 mm × 6 mm. The remaining four wells were used as a control (slide's polystyrene surface). Chitosan has the property to swell in wet conditions. Therefore, few drops of medium were poured into each well before inserting a ChM piece in a well. When a ChM piece swelled, it covered almost whole area of a well (8 mm × 8 mm). Following insertion of ChM, each chamber slide was sterilized by overnight UV exposure and subsequently 2-hour incubation with medium containing antibiotics. RSCs (20,000 cells/well) were seeded in complete RSCs culture medium and incubated for 8 hours, 1 day, 4, and 11 days in standard culture conditions. At each time point, cells were analyzed for cell adhesion, proliferation, and viability, and for expression of different proteins using a viability/cytotoxicity assay kit (Biotium Inc., USA) and immunostaining technique followed by observations in a phase-contrast and fluorescence microscope Leica AF6000 (Leica Microsystems, Mannheim, Germany).

### 2.4. Cell Adhesion and Proliferation

ChM and polystyrene surfaces were washed with PBS at each experimental time point to remove nonadhered cells. Numbers of adhered cells were determined by manual counting using a phase-contrast and fluorescence microscope. Cells were nuclear-stained with DAPI for 2 minutes at RT, mounted in a fluorescent mounting medium (Invitrogen, Paisley, UK), and visualized using a fluorescence microscope. Twenty fields (×10) were photographed at random per substrate. The cells, and their nuclei, contained in each field were counted using Adobe Photoshop Elements software. The mean number of nuclei per field of view (×10) was calculated for each time interval for each treatment and presented as a histogram showing the average nuclear count per field of view ±1 standard deviation (SD) versus time.

To determine the cell attachment to both surfaces, cells were counted after 8 hours. Average number of cells on the polystyrene substrate (positive control) was set to 100% and the average number of cells on the ChM surface was calculated as a percentage of the cells growing on the polystyrene surface for quantifying the percentage of cell adhesion and presenting as a histogram. The following formula was used to analyse the percentage of cell adhesion on ChM:

(1) %  cell  adherence(ChM)  =live  cells(ChM)live  cells(polystyrene)×100.

### 2.5. Cell Viability

RSCs viability was evaluated using a cell viability/cytotoxicity assay kit for live and dead cells in accordance with manufacturer protocol at each time point of the experiment. The kit includes two-color fluorescent stains: green fluorescence for live cells and red fluorescence for dead cells using two probes; calcein acetoxymethyl ester (calcein AM) stains live cells green and Ethidium homodimer III (EthD-III) stains dead and damaged cells red. After staining, RSCs were visualized using a fluorescence microscope and were photographed at random per well.

Percentages of cell viability were determined using the following formulas:

(2) $$\begin{array}{c} \%\;\text{live}\;\text{cells}=\frac{\text{live}\;\text{cells}}{\text{live}\;\text{cells}+\text{dead}\;\text{cells}}\times 100,\\ \%\;\text{dead}\;\text{cells}=\frac{\text{dead}\;\text{cells}}{\text{live}\;\text{cells}+\text{dead}\;\text{cells}}\times 100. \end{array}$$

### 2.6. Immunofluorescence Staining

RSCs were immunostained with antibodies against GFAP, PKC, isolectin, recoverin, and RPE65 for evaluating the expression of markers of different cells types. RSCs characteristics stability on ChM and polystyrene surfaces was evaluated by immunostaining for detecting the markers of epithelial (panCytokeratin), retinal stem (PAX6), and neural stem (nestin) cells as well as a marker of transdifferentiation towards fibroblast-like cells (alpha-SMA). In brief, cells were washed with PBS (3 × 5 min), fixed with methanol for 10 minutes at −20°C. At this step, the slides can be stored at −20°C in refrigerator. Cells were blocked for 1 hour in antibody blocking buffer (10% normal goat serum in PBS) at RT. Cells were then incubated overnight with different concentration (Table 1) of primary antibodies diluted in PBS at 4°C, then washed with PBS (3 × 5 min), and incubated with different concentration of secondary antibody (Table 1) diluted in PBS for 1 hour at RT. The cells were also costained with a 1 : 500 dilution of DAPI in PBS for 2 minutes, mounted using a fluorescence mounting medium (DAKO North America, Inc, Carpinteria, CA, USA), and observed under a fluorescence microscope Leica AF6000.

### 2.7. Statistical Analysis

All experiments were repeated three times to check the reproducibility of the trends observed. The data were obtained from repetition of the experiments subjected to the statistical analysis through Microsoft Excel software. Average, standard deviation (SD), and *P* values were calculated. Statistical significance was set at *P* < 0.05 and *P* < 0.01.

## 3. Results

### 3.1. RSCs Spheres, Morphology, Pigmentation, and Differentiation Potential

**Figure 1:**
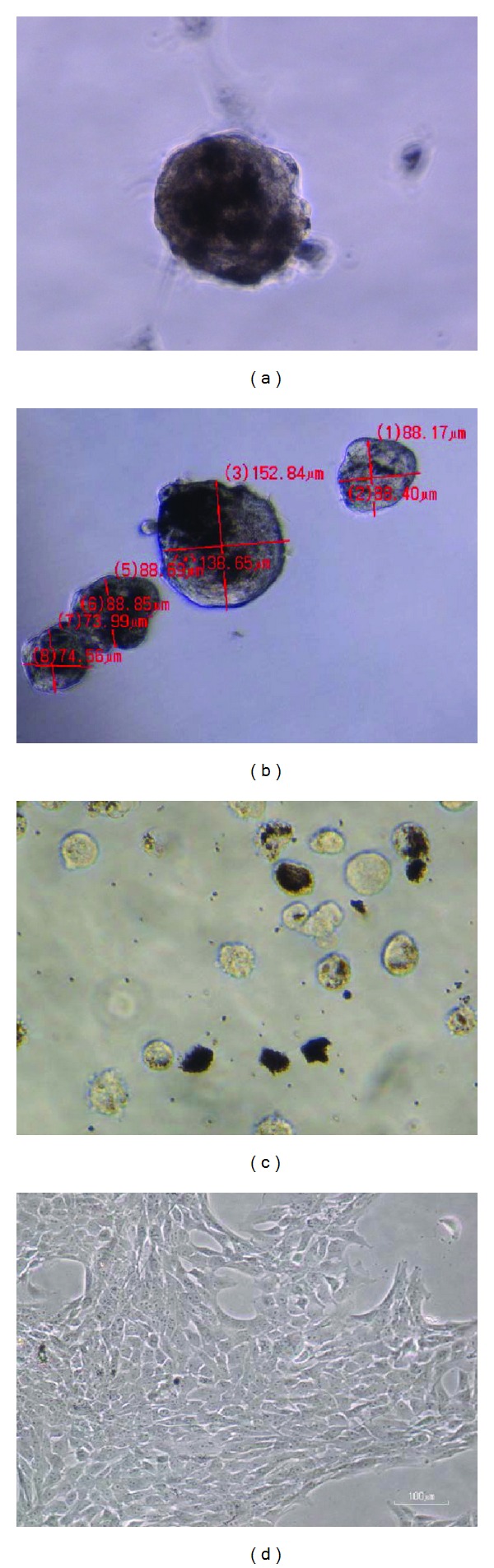
Morphology and pigmentation of RSC spheres and RSCs in 10x microscope field observation. (a) Free floating RSC sphere at 1 week, (b) RSC spheres of different sizes at day 18. (c) Pigmented and nonpigmented RSCs at day 24. (d) Confluent RSCs layer with epithelial-like to fibroblast-like cell morphology. (a), (b) and (c) are taken in 10x microscopic field but trimmed down to increase the size of image to show clearly cells in neurosphere (a), scale bar (b) and pigments in cells (c).

Ciliary margin isolated RSCs began to form floating cell spheres after one week in standard culture medium (Figure 1(a)). These RSC spheres were of variable sizes varying from 74 *μ*m × 73 *μ*m to 138 *μ*m × 152 *μ*m, and with pigmentations as observed in 10x microscope field at 18 days (Figure 1(b)). After 24 days, all RSC spheres were collected, washed with PBS and dissociated using trypsin-EDTA solution, and cultured in complete standard RSCs medium. The dissociated RSC spheres showed the presence of the pigmented and nonpigmented cells (Figure 1(c)). These cells adhered to the surface of culture flask and formed monolayer. Cells of adhered confluent RSCs layer acquired variable morphology varying from epithelial-like to fibroblast-like (Figure 1(d)).

**Figure 2:**
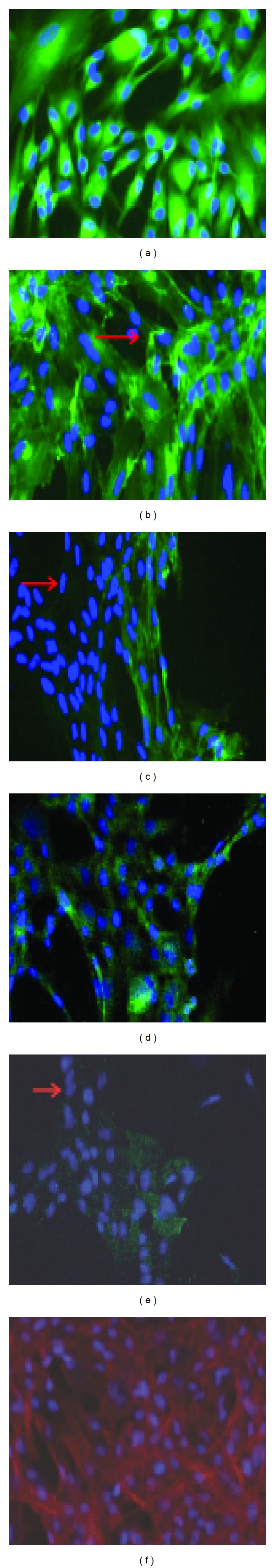
RSCs cultivated in standard culture medium supplemented with 10% FBS expressed the proteins (green) of different retinal cell types. Expression of protein; (a) GFAP (astrocyte), (b) isolectin (microglial cells), (c) PKC (rod bipolar cells), (d) recoverin (photoreceptor), (e) RPE65 (RPE cells). Rhodamine-phalloidin and DAPI staining showed actin (red) and nucleus (blue). The arrow showed the cells which are not expressing the proteins studied.

Immunostaining results showed that RSCs expressed the proteins related to different retinal cell types. Almost all RSCs expressed GFAP (astrocytes, Figure 2(a)) and recoverin (photoreceptor, Figure 2(d)) isolectin (microglial cells, Figure 2(b)) proteins; however, most of the cells lacked the expression of PKC (rod bipolar cells, Figure 2(c)) and RPE65 (RPE cells, Figure 2(e)) proteins.

### 3.2. RSCs Adhesion and Proliferation

**Figure 3:**
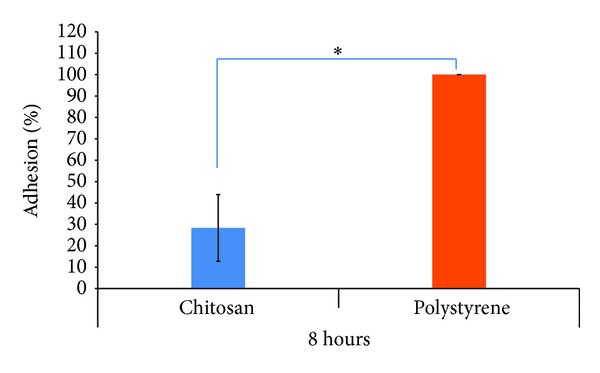
Percentage of RSCs adhered on surfaces, ChM and polystyrene, at 8 hours. The data presents the mean number of cells (phase-contrast microscopy as well as nuclear counts of cells, assuming 1 nucleus per cell) per field (×10) attached to ChM surface as a percentage of the control (polystyrene) ±1 SD, at 8 hours. The histogram confirmed that ChM surface is less favourable for RSCs adhesion than polystyrene surface. Single asterisk (*) represents the significant *P* value between percentage of cell adhesion on chitosan and polystyrene surface. Statistical significance is adjusted at *P* < 0.05 (*).

**Figure 4:**
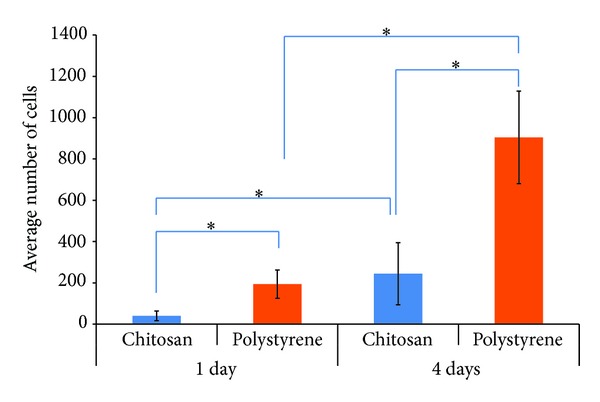
Average number of RSCs grown on surfaces, ChM and polystyrene, at days 1 and 4 determined using cell viability/cytotoxicity assay kit. The data presents the average number of cells per field (×10) attached to both surfaces ±1 SD, at days 1 and 4. The histogram confirmed that ChM surface is less favourable for RSCs growth than polystyrene surface. Single asterisk (*) represents the significant *P* value between average number of cells on chitosan and polystyrene surface at days 1 and 4. Statistical significance is adjusted at *P* < 0.05 (*).

**Figure 5:**
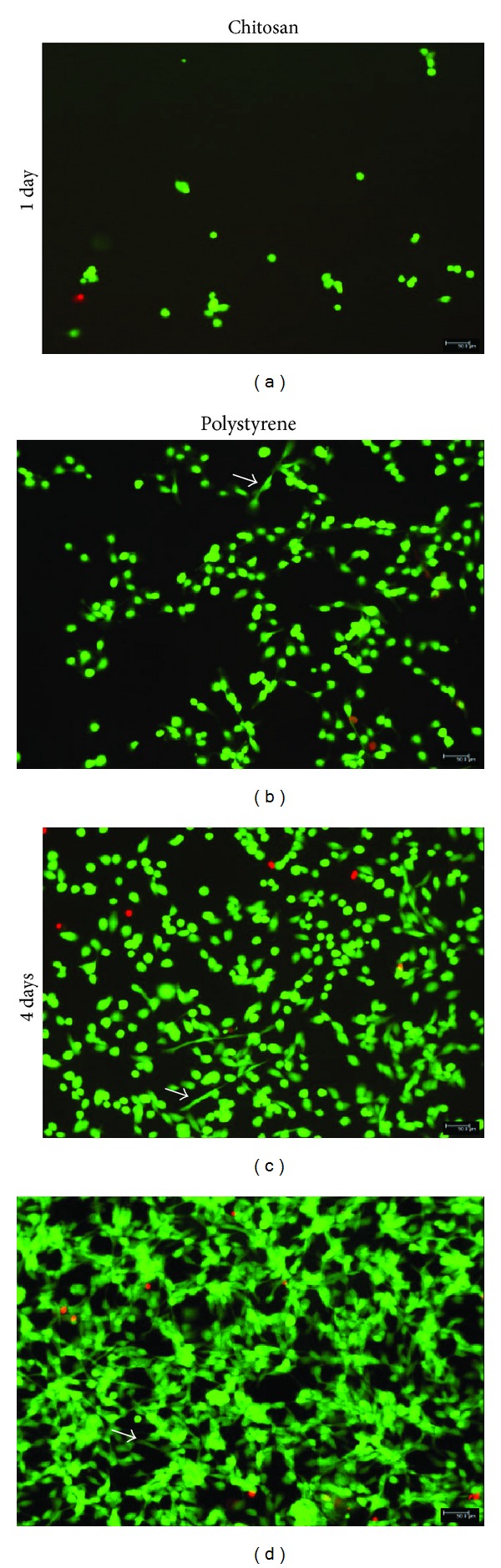
Viability and morphology of RSCs on ChM and polystyrene surfaces detected using cell viability/cytotoxicity assay kit. The green fluorescence represents live cells and red fluorescence represents dead cells. (a) RSCs on ChM surface at day 1. (b) RSCs on polystyrene surface at day 1. (c) RSCs on ChM surface at day 4. (d) RSCs on polystyrene surface at day 4. The figures are representative figures of various photos of RSCs grown on both surfaces and subsequently stained for live and dead cells analysis. White arrows show fibroblast-like morphology.

Very few RSCs adhered faintly on ChM surface during 4-5 hours. At 8 hours, RSCs adhered significantly less (28%) on ChM than that observed on polystyrene (Figure 3). RSCs grew less at day 1 (average 40 cells/microscopic field) and at day 4 (average 244 cells/microscopic field) on ChM in comparison to polystyrene (194 and 904 cells/microscopic field, resp.). Although RSCs number increased on both surfaces at day 4 in comparison to day 1 (Figures 4 and 5), the number of cells on polystyrene surface was significantly higher (*P* < 0.05) than ChM surface at both time points (Figures 4 and 5). RSCs growth on ChM surface increased with time but it was always less than the RSCs growth on polystyrene (Figure 4).

### 3.3. RSCs Viability and Morphology

**Figure 6:**
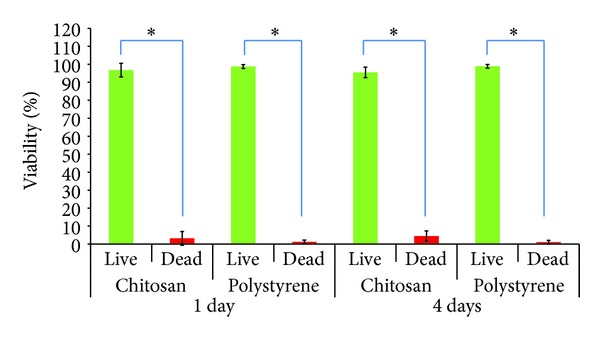
Viability of RSCs on ChM and polystyrene surfaces at days 1 and 4. Cells were quantified using cell viability/cytotoxicity assay kit on both surfaces. The data are presented as percentage of viable and dead cells per field (×10) ±1 SD following the formulas written in Section 2. The histogram showed that both surfaces maintained over 90% viability of cells despite differences in the adherence and growth. Single asterisk (*) represents the significant *P* value between live and dead cells grown on chitosan and polystyrene surface at days 1 and 4. Statistical significance is adjusted at *P* < 0.05 (*).

Viability/cytotoxicity assay showed that very few dead cells (1–7) were present in each photo of 10x microscopic field taken for both surfaces at days 1 and 4 (Figure 5). Statistical analysis showed that this was significantly very low (*P* < 0.05) in comparison to high number of living cells observed in each field (Figures 5 and 6). Further percentage viability analysis using the formulas confirmed that RSCs maintained above 95% viability during growth on both surfaces at each time point of the experiment (Figure 6).

Phase contrast microscopy (data not shown) as well as cell viability/cytotoxicity assay kit showed that, at day 1, RSCs were rounded on ChM surface while RSCs on polystyrene surface began to take fibroblast-like shape (Figures 5(a) and 5(b)). At day 4, few RSCs on ChM surface also began to adapt fibroblast-like shape but it was significantly low (*P* < 0.05) than that observed on polystyrene surface (Figures 5(c) and 5(d)).

### 3.4. Characteristics of RSCs Grown on ChM

**Figure 7:**
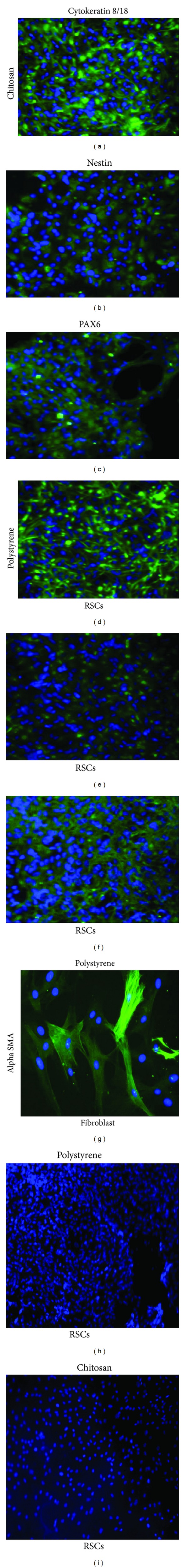
Immunostaining of proteins expressed by RSCs grown on ChM and polystyrene surfaces for 11 days. Expression of protein: cytokeratin 8/18 on ChM (a) and polystyrene (d), nestin on ChM (b) and polystyrene (e), PAX6 on ChM (c) and polystyrene (f), alpha-SMA on polystyrene (h) and ChM (i). Alpha-SMA protein expression of fibroblasts (g). Polystyrene surface and fibroblast were used as controls; (g) is taken in 20x microscopic field and the rest are in 10x microscopic field.

Immunostaining results showed that RSCs grown on ChM and polystyrene surfaces expressed cytokeratin 8/18, PAX6 as well as nestin proteins at day 11 (Figures 7(a)–7(f)). Alpha-SMA expression in the cells grown on ChM as well as polystyrene surfaces was not detected at the same time period (Figures 7(h) and 7(i)) while the expression was detected in fibroblast (Figure 7(g)) used as a control.

## 4. Discussion

Although adult mammalian retina retains RSCs in quiescent form in *in vivo* ciliary margin, it lacks the self-regeneration process in response to *in vivo* damages. Nevertheless, they have capacity to self-renewal and to differentiate into different retinal cell types *in vitro* conditions. Cell transplantation seems one of the most feasible approaches to repair the retinal damages, but only some of them achieved partial success [2, 3].

Porcine eye resembles human eye in many properties such as similar size, anatomy, and histology. Furthermore, retinal development in pig eye shows substantial similarity to human retinal development. These characteristics make pig eyes and their retinal cells an ideal model for performing preclinical tests [25, 26].

The data obtained in this study indicates that RSCs can be isolated and cultured *in vitro* successfully in appropriate culture conditions. Isolated RSC began to form pigmented clumps of the cells (RSC sphere), and, after 1 week, few RSC spheres could be observed floating in the culture medium. At day 18, RSC spheres were found in variable sizes varying from 74 *μ*m × 73 *μ*m to 138 *μ*m × 152 *μ*m. This confirmed that RSCs in culture medium as well as in each sphere were proliferating and growing. Microscopic observations showed that RSC spheres contained pigments. At day 24, RSC spheres were collected and trypsinized when it seemed that most of the RSC spheres achieved suitable size for performing further study with them. The microscopic evaluation after disintegration of RSC spheres using trypsin-EDTA confirmed the presence of pigmented and nonpigmented RSCs cells in spheres. These pigmented and nonpigmented RSCs could be different in their proliferative and differentiation potential and it is under further investigation.

The controversial reports [27, 28] questioning the RSCs existence in ciliary margin supported further RSCs characterization using immunostaining techniques. The result showed that RSCs expressed proteins, those belong to different retinal cell types. Cytokeratin 8/18 detection in these cells confirmed the epithelial characteristic but RSCs also expressed PAX6, a retinal stem cell nuclear and cytoplasmic protein, nestin, a neural progenitor cell protein, GFAP, an astrocytes protein, recoverin, a photoreceptors protein, isolectin, a microglial cell protein, PKC, a rod bipolar cell protein, and RPE65, a RPE cell protein [9, 29]. It also cannot be avoided that two or more types of cells share a single protein such as Muller and RPE cells expressing CRALBP protein. PAX6 protein is not only a retinal stem cell marker. This transcription factor is expressed in mature retinal cells such as amacrine cells and also in nonretinal cells from different part of the central nervous system. It was also found that, in some cases, almost all the cells expressed the specific proteins (GFAP, recoverin, and isolectin) and, in another case, very few cells expressed the specific proteins (PKC and RPE65). However, cell quantification and combination of different staining in further study will provide a clear image of percentage of cell population expressing a specific protein. Thus, presence of proteins of differentiated and undifferentiated cells confirmed that RSCs are somewhere in midway of undifferentiation to differentiation pathway. It seems that they arrived at an immature stage from where the differentiation to a specific retinal cell (RPE, photoreceptor, etc.) starts. Further stimuli need to decide the fate of these immature cells, which could be provided by *in vivo* retinal environment after cell transplantation [30]. Due to this reason, an adequate support is required that can carry RSCs without alternating their behaviors as well as the support must increase the possibility to deliver RSCs into retina.

RSCs were faintly attached to ChM surface than to polystyrene surface in the first few hours. However, with time, RSCs adherence on surfaces increased significantly and, at 8 hours, 28% of RSCs were attached on ChM surface. This showed that RSCs adapted the unknown internal changes to favour their attachment on ChM surface along with time. Cell viability/cytotoxicity analysis at days 1 and 4 showed that RSCs grew well on ChM surface with time but significantly less than that grew on polystyrene surface. The difference of the cell numbers on surfaces (ChM versus polystyrene) increased significantly along with time. RSCs maintained same viability (<90%) on ChM surface as observed on polystyrene surface. The reason behind low RSCs adherence and proliferation on ChM surface is unknown but it confirms the role of structure and property of chitosan molecules acting as biophysical or biochemical cues for cultivation of RSCs on ChM surfaces.

Image analysis showed that RSCs were rounded at 8 hours (figure not shown) and they started to adopt fibroblast-like shape at day 1. But the cells with fibroblast-like shape were significantly less on ChM than polystyrene surface. On polystyrene surface, RSCs started to adopt fibroblast-like shape in early hours (day 1), and, at day 4, numerous cells with fibroblast-like shape could be observed. This confirmed that polystyrene surface is more favourable for RSCs for adopting fibroblast-like shape. Fibroblast-like shape indicates the cells under transition to another type of cells such as epithelial cells or fibroblast or specific retinal cells; therefore, the results confirmed that ChM promotes the RSCs transition.

Immunofluorescence study of RSCs confluent layer at day 11 showed that RSCs expressed cytokeratin8/18, PAX6, and nestin proteins on both surfaces. As previously mentioned, cytokeratin 8/18 protein is an epithelial cell protein, PAX6 is a retinal stem cell protein, and nestin is a neural stem cell protein. These proteins were selected for immunostaining in this study because the results can provide preliminary data about the RSCs differentiation direction to retinal, nonretinal, or epithelial cells on the different surfaces. RSCs gown on both surfaces showed the expression of these proteins, confirming that RSCs maintained the characteristics on ChM as observed on polystyrene. Anti-alpha-SMA antibody is used to detect transdifferentiation of cells into fibroblast. Alpha-SMA protein expression was detected in RSCs grown neither on ChM surface nor on polystyrene surface; however, it was detected in fibroblast used as a control. This further confirmed that RSCs maintained similar characteristics on ChM and polystyrene surfaces. The level of expression of these proteins, detecting other proteins such as proteins for type of retinal neurons differentiated in such conditions and cell quantification, is still an issue of investigation. Thus, RSCs growing on ChM are under cellular transition, forming fibroblast-like shapes, but not driven towards differentiation to fibroblast. It could be stimulated to differentiate into another type of cells which is still under investigation.

Chitosan is a FDA approved biomaterial used in clinics for various purposes due to its safe, tolerable, and biocompatible nature. Although polystyrene supports sufficiently the cellular behavior as well as growth, it cannot be alternative of chitosan because it does not have similar nature as chitosan contains. It is well known that commercially available polystyrene dishes are treated to release or activate chemical groups responsible for cell adhesion and proliferation; therefore, such treatments, if feasible, with ChM, would improve RSCs behavior on ChM surface. However, as previously mentioned, the fate of RSCs to differentiate into different retinal cell type could depend on the stimulus obtained in retinal environment. Therefore, ChM could be applicable in RSCs transplantation as a cell carrier because it would not affect the cell behaviour. Additionally after RSCs transplantation, poor cell growth and adhesion support the proliferating RSCs to migrate and integrate into retina and differentiate into appropriate cell types in accordance with the stimulus obtained. However, a further *in vivo* study needs to prove this concept including many other issues such as that chitosan scaffolds with retinal progenitors upon transplantation into the retina would break down into long chain sugars and would take a very long time to degrade. Additionally, this cannot be avoided that ciliary margins are not a realistic source for RSCs because, in practical, a biopsy of an adult retina will promote its further degeneration. However, this proof of the concept *in vitro* and *in vivo* would support utilizing the ChM as a cell carrier for transplanting those cells that show the RSCs-like phenotype and need a further stimuli form retinal environment.

## 5. Conclusion

RSCs express proteins associated with epithelial, stem, and different retinal cells confirming its potentiality to move towards undifferentiated or differentiated cells depending on stimulus received. RSCs adhere and grow poorly on ChM surface but they maintain similar viability and characteristics on ChM and polystyrene surfaces. Thus, ChM application as a RSCs carrier in cell transplantation increases the feasibility that proliferating RSCs can migrate, differentiate, and integrate in retina. However, further *in vivo* studies are required for strengthening the conclusion.

## Figures and Tables

**Table 1:** List of antibodies used in the study.

Molecular marker	Antibody	Source	Working dilution
Cytokeratin 8/18	Mouse monoclonal	Abcam, Cambridge, UK	1 : 100
Nestin	Mouse monoclonal	Abcam, Cambridge, UK	1 : 100
PAX6	Rabbit polyclonal	Covance, Emeryville, CA, USA	1 : 100
Alpha-smooth muscle actin (alpha-SMA)	Mouse monoclonal	Abcam, Cambridge, UK	1 : 200
Glial fibrillary acidic protein (GFAP)	Rabbit polyclonal	DakoCytomation Inc., USA	1 : 200
Isolectin	Mouse monoclonal	Sigma-Aldrich	1 : 100
Protein kinase C, *α* isoform (PKC*α*)	Rabbit polyclonal	Santa Cruz Biotechnology, Inc., USA	1 : 50
Recoverin	Rabbit polyclonal	Millipore, CA, USA	1 : 100
RPE65	Mouse monoclonal	Novus biological, UK	1 : 100
